# Influence of taxonomic resolution on mutualistic network properties

**DOI:** 10.1002/ece3.6060

**Published:** 2020-03-06

**Authors:** Estelle Renaud, Emmanuelle Baudry, Carmen Bessa‐Gomes

**Affiliations:** ^1^ Ecologie Systématique Evolution CNRS AgroParisTech Université Paris‐Saclay Orsay France

**Keywords:** connectance, modularity, nestedness, networks, plant–pollinator, robustness, taxonomic resolution

## Abstract

1. Ecologists are increasingly interested in plant–pollinator networks that synthesize in a single object the species and the interactions linking them within their ecological context. Numerous indices have been developed to describe the structural properties and resilience of these networks, but currently, these indices are calculated for a network resolved to the species level, thus preventing the full exploitation of numerous datasets with a lower taxonomic resolution. Here, we used datasets from the literature to study whether taxonomic resolution has an impact on the properties of plant–pollinator networks.
2. For a set of 41 plant–pollinator networks from the literature, we calculated nine network index values at three different taxonomic resolutions: species, genus, and family. We used nine common indices assessing the structural properties or resilience of networks: nestedness (estimated using the nestedness index based on overlap and decreasing fill [NODF], weighted NODF, discrepancy [BR], and spectral radius [SR]), connectance, modularity, robustness to species loss, motifs frequencies, and normalized degree.
3. We observed that modifying the taxonomic resolution of these networks significantly changes the absolute values of the indices that describe their properties, except for the spectral radius and robustness. After the standardization of indices measuring nestedness with the *Z*‐score, three indices—NODF, BR, and SR for binary matrices—are not significantly different at different taxonomic resolutions. Finally, the relative values of all indices are strongly conserved at different taxonomic resolutions.
4. We conclude that it is possible to meaningfully estimate the properties of plant–pollinator interaction networks with a taxonomic resolution lower than the species level. We would advise using either the SR or robustness on untransformed data, or the NODF, discrepancy, or SR (for weighted networks only) on *Z*‐scores. Additionally, connectance and modularity can be compared between low taxonomic resolution networks using the rank instead of the absolute values.

## Graphical abstract

Using plant–pollinator networks from the literature, we show that commonly used network metrics can be meaningful on networks with lower‐than‐species resolution. Spectral radius and robustness to taxa loss can be used at species, genus, and family levels. The relative values of all metrics studied are strongly conserved at different taxonomic resolutions.

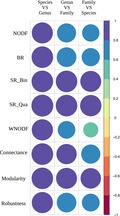

## 1 INTRODUCTION

The study of species interactions has always been central in ecology. Such interactions have historically been examined by focusing on two interacting species, but in recent years, the marked increase in the amount of biological information available and the development of novel approaches and tools have placed a new focus on the study of interaction networks (Proulx, Promislow, & Phillips, 2005). Ecological networks may provide important insights that cannot be gained when species are studied in isolation. They currently play a central role in key aspects of ecological theory such as the long‐standing question of the relationship between complexity and stability in ecosystems (Montoya, Pimm, & Solé, 2006; Thébault & Fontaine, 2010) or the interplay between interspecific competition and ecological niche (Bastolla et al., 2009). Ecological networks are also powerful tools for applied ecology, as they can be used to monitor the impact of biological perturbations on an ecosystem or the efficiency of restoration programs (Kaiser‐Bunbury & Blüthgen, 2015; Kaiser‐Bunbury et al., 2017; Memmott, 2009).

Most studies on ecological networks have focused on three main categories of networks defined according to the type of species and their interactions: food webs, parasitoid host interaction networks, and more recently, mutualist interaction networks (Ings et al., 2009). In this paper, we concentrate on the case of the mutualistic networks linking plants and pollinators, which have attracted particular attention in recent years. Indeed, pollinators have an essential ecological function, namely the pollination function, which is threatened in many parts of the world by the sharp decline in pollinators on account of the many threats that they face (Goulson, Nicholls, Botías, & Rotheray, 2015). Such a decline in pollinator populations may harm both wild biodiversity and agricultural productivity (Garibaldi et al., 2013).

The use of a network makes it possible to synthesize in a single object the species and interactions linking them and thus constitute the community of species (Delmas et al., 2019). It thus becomes possible to use the many methods developed to study ecological networks to describe their structure and properties using different indices (Lau, Borrett, Baiser, Gotelli, & Ellison, 2017). One structural characteristic that has received particular attention in the study of plant–pollinator networks is nestedness (Bascompte, Jordano, Melián, & Olesen, 2003; Table 1). A nested network is characterized by the extent to which interactions of less‐connected species form subsets of the interactions of more‐connected species. Other frequently examined structural characteristics of mutualistic networks are connectance, the proportion of realized interactions among all possible ones, and modularity, that is, the extent to which linked interactions between pollinators and plants are organized into delimited modules, as well as motifs, which are subnetworks representing the interactions between a given number of taxa (Milo et al., 2002). These properties have been associated with the ecosystem's resilience to perturbations (Soares, Ferreira, & Lopes, 2017). It has, for example, been shown that high levels of connectance, modularity, and nestedness promote both the structural and dynamic stability of mutualist interaction networks (Vanbergen, Woodcock, Heard, & Chapman, 2017).

**Table 1:** Commonly used network indices

Index	Matrix^a^	Nature of the index, per network	Representation
Nestedness			
NODF	B	Extent to which interactions of less‐connected species form subsets of the interactions of more‐connected species	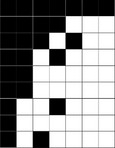
BR	B		
SR	B W		
WNODF	W		
Connectance	B	Proportion of realized interactions among all possible ones	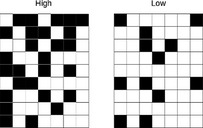
Modularity	W	Extent to which interactions between pollinators and plants are organized into delimited modules	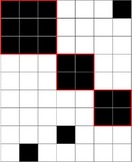
Robustness	B	Speed at which plant taxa disappear as pollinator taxa disappear	
Normalized degree	B	Connectance of each taxa (this is the only index calculated per taxa and not per network)	
Motif frequency	B	Frequency of each of the 17 kinds of motifs that can link up to 5 taxa between them	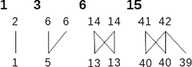

Abbreviations: BR, discrepancy; NODF, nestedness index based on overlap and decreasing fill; SR_Bin, spectral radius calculated on binary (absence/presence) matrices; SR_Qua, spectral radius calculated on weighted (abundance) matrices; WNODF, NODF calculated on weighted matrices.

a B and W indicate an index calculated on binary and weighted matrices, respectively.

A large number of datasets on plants and their pollinators have been collected to date. However, given the large number of pollinator species potentially present in a community, as well as the relative difficulty in identifying some of these pollinators at the species level, a significant portion of the collected datasets has a taxonomic resolution lower than the species level. For a given research effort, there is therefore a trade‐off between the quantity of possible identifications and the taxonomic accuracy of these identifications, which makes it difficult to produce large or numerous sets of data identified down to the species level. An extreme point in this regard is the datasets provided by citizen science programs for pollinators (Toomey & Domroese, 2013) such as the Spipoll program in France (Deguines, Julliard, Flores, & Fontaine, 2012), which generally allow very large datasets to be collected, although their taxonomic accuracy does not generally extend to the species level (Dickinson, Zuckerberg, & Bonter, 2010; Kremen, Ullman, & Thorp, 2011).

Currently, network analyses are performed on networks with varying levels of taxonomic precision, which makes comparisons between studies or even sites of the same studies potentially invalid, because we do not know how taxonomic resolution influences the indices of those networks, nor how they should be interpreted. If possible, it would, however, be interesting to use network analyses on such datasets in order to fully exploit the information contained therein and allow comparisons with other studies. Here, we sought to establish whether taxonomic resolution has an influence on the architecture and properties of a mutualistic network estimated using several indices. We used a set of 41 plant–pollinator networks from the literature and compared their index values at three different taxonomic resolutions: species, genus, and family. We showed that for a given network, changing the taxonomic resolution usually significantly changes the value of most indices. We also show that after the standardization (with the *Z*‐score, using null models) of the indices measuring nestedness, these three indices are no longer differed significantly at different taxonomic resolutions. We also used another normalization measure for one nestedness index (NODF) called NODFc and show that this measure is robust to a lower taxonomic resolution (Song, Rohr, & Saavedra, 2017). Additionally, we showed that among the set of 41 networks, the relative value of a given network for a given index is well conserved across different taxonomic resolutions, particularly between the species and genus levels.

## 2 MATERIALS AND METHODS

### 2.1 Overview

We used plant–pollinator networks from the literature (Vázquez, Goldberg, & Naik, 2003) determined to the level of species. For each species‐level network, we deduced the equivalent network at the genus and family levels. We then calculated several indices commonly used to estimate mutualistic network properties for each of these networks and then compared their values across taxonomic resolutions. Data manipulation and analysis were conducted with the R language (R version 3.2.3, 2015‐12‐10). The script used for those results is accessible here: https://gitlab.com/EstelleRenaud/taxonomic_influence_network_properties

### 2.2 Network indices

We selected frequently used indices that describe various properties of interaction networks, namely nestedness, connectance, modularity, motifs, and robustness. Given the particular importance of generalist pollinator species in maintaining plant–pollinator networks (Martín González, Dalsgaard, & Olesen, 2010), we also added one index calculated at the species level, that is, the normalized degree.

The characteristics of these indices are summarized in Table 1.

To estimate nestedness, no unique index has been established to date as consensual, which led us to use four indices: nestedness index based on overlap and decreasing fill (NODF; Almeida‐Neto, Guimarães, Guimarães, Loyola, & Ulrich, 2008), spectral radius (SR; Staniczenko, Kopp, & Allesina, 2013), discrepancy (BR; Brualdi & Sanderson, 1999), and NODF for weighted matrices (WNODF; Almeida‐Neto & Ulrich, 2011). NODF and BR are indices for binary matrices, while WNODF is adapted to weighted matrices and SR can be used for both. The values for NODF, WNODF, BR, and SR were calculated using the *Falcon* package (Beckett, Boulton, & Williams, 2014) for R. Furthermore, BR was calculated using the method presented in Brualdi and Sanderson (Brualdi & Sanderson, 1999): it is the minimal number of differences with a perfectly nested matrix with the same size, number of links, and column (or row) sums as the real network. The SR of a network is thus the largest of its matrix eigenvalues (Staniczenko et al., 2013).

We used five additional indices. Four of them—connectance, robustness, motifs, and normalized degree—are calculated on the presence/absence matrices, whereas modularity is calculated on frequency matrices. Network connectance was calculated as the sum of links divided by the number of cells in the matrix. Network modularity was measured according to the Beckett algorithm DIRTLPAwb+ (Beckett, 2016), which aims to estimate the modularity of the network using three steps. The first uses label propagation to obtain a locally maximized modularity (bottom‐up); the second agglomerates the modules found in the first step if it allows for an increased modularity; the third repeats these steps until modularity can no longer be increased. DIRTLPAwb+ then randomizes the initial labeling of nodules multiple times and returns the result with the greatest modularity score. Modularity itself was then calculated as the modularity M proposed by Newman (Newman, 2006). Following Burgos et al. (2007), network robustness was measured as the area under the attack tolerance curve, defined as the speed at which plant taxa disappear as pollinator taxa disappear. Basically, as pollinator taxa disappear, plants that rely exclusively on them (according to the network) also disappear, thus creating a curve in which the percentage of remaining plants depends on the percentage of remaining pollinators. This method assumes that preferences are static, that is, that plants that rely on one given pollinator taxon will not be able to switch to another pollinator if it disappears. Normalized degree is calculated for each taxon in a network as the sum of the links of that taxon scaled by its number of potential partners. Because the normalized degree index produces one value per taxon, for each matrix, we chose to characterize matrices by their quartile values in order to accommodate differently skewed distributions of the normalized degrees between networks. The R package *bipartite* (Dormann, Gruber, & Fründ, 2008) was used to calculate the normalized degree, robustness, modularity, and connectance of the network. Motifs were compared using the frequency of the 17 different motifs that involve up to five different taxa, which we calculated using the *mcount* function of the *bmotif* package (Simmons et al., 2019). The frequency of each motif was calculated as the number of times a given motif occurs in the network, divided by the number of motifs of the same size (involving the same number of taxa) that occurs in the network.

Additionally, because most nestedness indices are known to be sensitive to the size (number of rows and columns) and fill (number of nonzeros) of the input matrix (Rodríguez‐Gironés & Santamaría, 2006)—two properties that are modified when the taxonomic resolution is changed—we performed standardization with *Z*‐scores for nestedness indices (Ulrich & Almeida‐Neto, 2012). These are calculated as the difference between the observed index value and the value expected under a null model divided by the standard deviation under this null model. *Z*‐scores were obtained by calculating 500 null models of each matrix and comparing the resulting mean value to that calculated for the matrix. As the null models take into account the size of the matrix, this minimizes the possible effect of size on the index values. We used the *Falcon* package to calculate the *Z*‐scores. For the binary indices (BR, NODF, SR), we followed Bascompte et al. (2003) and used null models obtained from 500 iterations of the DD (degreeprobable–degreeprobable) model. This model is intermediate in terms of constraints on row and column totals (part of the class termed “PP,” proportional to both row totals and column totals; Strona, Ulrich, & Gotelli, 2018), with one extreme being the fixed–fixed model that is susceptible to type II errors (Gotelli, 2000) and the other the equiprobable–equiprobable model that is susceptible to type I error (Wright, Patterson, Mikkelson, Cutler, & Atmar, 1997). This model has statistically determined elements following the degree distribution of the initial matrix as *p_ij_* = 1/2*(*d_j_*/*r* + *k_i_*/*c*), where *p_ij_* is the probability of assigning a 1 to the *i*th row and *j*th column, *d_j_* is the column degree of the *j*th column, *k_i_* is the row degree of the *i*th row, and *r* and *c* are the respective numbers of rows and columns. For the weighted indices (WNODF, SR), we used two kinds of null models, as no null model has been established as more suited to WNODF or SR yet: The first set of null matrices is obtained from 500 iterations of the row and column total average model (introduced in the Falcon software) that averages two matrices: a matrix created conserving the row totals and redistributing a random portion of that total to each element of a given row, and a matrix following the same principle with the column totals. The second kind is Patefield's historical r2dtable model, implemented with the null model function (option “r2dtable”) of the bipartite R package (Dormann et al., 2008). We also generated 500 matrices under that model.

Finally, because *Z*‐scores for NODF have been criticized for their sensitivity to connectance and number of taxa (Song et al., 2017), we used the normalization proposed by Song et al. as NODF_c_ = NODF_n_/(C*log(*S*)), where *C* is the connectance, *S* is the geometric mean of plants and pollinators in the network, and NODF_n_ = NODF/max(NODF), where max(NODF) is the maximal NODF value that could be attained in a network with the same number of rows, columns, and links as the original network. Max(NODF) was calculated using the maxnodf R package (Hoeppke, 2019).

### 2.3 Pollination networks

We extracted all plant–pollinator interaction networks from the Interaction Web Database (Vázquez et al., 2003). All networks were issued from previously published data (Table S1). We only kept matrices for which taxa determination was possible using the taxize package; that is, valid taxonomic names resolved at the genus or species level. In some cases, we replaced old taxonomic names by a current valid synonym. We also only kept matrices that dealt with several families, which left us with a dataset of 41 matrices, 10 of which were binary (presence/absence) matrices. The remaining 31 were weighted according to the frequency of the visitation or a proxy of that frequency. The number of taxa in the matrices varied from seven to 135 for plants, and 12 to 144 for pollinators.

We then used the taxize package (Chamberlain & Szöcs, 2013; version 0.9.0) from R to extract from the taxonomic information supplied by the authors the taxonomic affiliation from the superior ranks. Only the identification from the species, genus, family, and order ranks was retained, as these were the ranks most often known for all observations. The database GBIF (GBIF, 2018) was used as a reference.

We transformed each of the 41 previously described matrices into interaction matrices determined at the species level by keeping only the observations (within each network) for which both the plant and pollinator were determined to the species level. From these species‐level matrices, we deduced the genus‐level and then the family‐level matrices.

### 2.4 Statistical tests

To examine the influence of the taxonomic level on the structure of a given matrix, we compared the values of the indices for Species‐level matrices and Genus‐level matrices, Genus‐level matrices and Family‐level matrices, and Species‐level matrices and Family‐level matrices, using a one‐way analysis of variance. Post hoc tests were performed with a Bonferroni correction, using the built‐in pairwise.*t*.test R function, with the “paired” option. We also performed the same analyses after standardizing nestedness values using *Z*‐scores.

To investigate whether an index was useful for comparing different observed matrices, we performed a nonparametric correlation test (cor.test on R) to calculate both the value and significance of Spearman's rho for a given index in Species‐level matrices, Genus‐level matrices, and Family‐level matrices. This allowed us to test whether the relative ranks of this index's values were significantly correlated between one taxonomical level and another.

## 3 RESULTS

**Figure 1:**
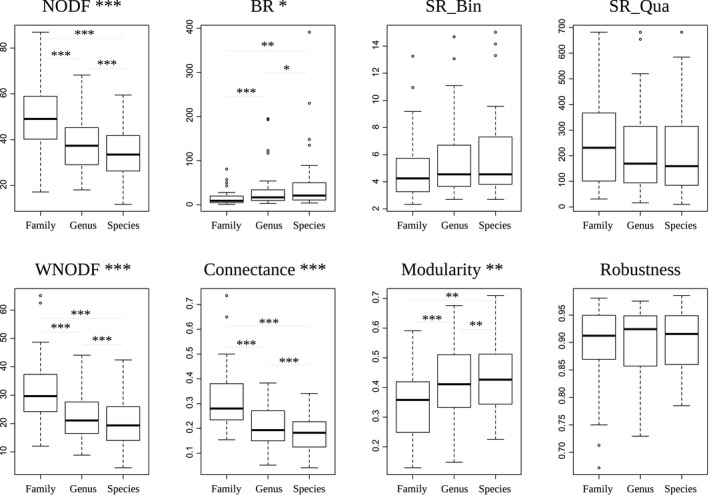
Untransformed index value distribution for species (S)‐, genus (G)‐, and family (F)‐determined matrices. From left to right and top to bottom: NODF, BR, SR_Bin, Robustness, Connectance, WNODF, SR_Qua, Modularity. BR, discrepancy; NODF, nestedness index based on overlap and decreasing fill; SR_Bin, spectral radius calculated on binary (absence/presence) matrices; WNODF, NODF calculated on weighted matrices. 0.001 < *** < 0.01 < ** < 0.05; *Next to the index name reflects the result of the ANOVA test, on the graph itself reflects the results of the post hoc paired *t* test, with a Bonferroni correction

For each of the species‐, genus‐, and family‐determined matrices, we obtained a set of values per index for the species‐determined matrices, and another set for genus‐ and family‐determined matrices. We compared these using a one‐way analysis of variance. Most indices show a significant effect of taxonomic level on their value (Table 2 and Figure 1; results for normalized degrees and motifs frequencies are presented in the Appendix A). Robustness and SR (both on binary and on weighted matrices) are the only indices without any significant influence of taxonomic level on their values. This indicates that two matrices cannot be directly compared with most indices if they are not at the same level of taxonomic resolution. The same is true with family versus genus comparisons, and family versus species comparisons.

**Table 2:** Results of one‐way analysis of variance comparing index values at three different taxonomic resolutions

Indices	*df*	*F*	*p*‐Value	Post hoc test results
Nestedness				
NODF	2, 120	15.03	1.496e−05	All levels differ significantly
BR	2, 120	4.2035	.0172	All levels differ significantly
SR_Bin	2, 120	1.1573	.3178	
SR_Qua	2, 90	0.5765	.5639	
WNODF	2, 90	11.626	3.229e−05	All levels differ significantly
Connectance	2, 120	20.237	2.67e−08	All levels differ significantly
Modularity	2, 90	5.2213	.007155	All levels differ significantly
Robustness	2, 120	0.0831	.9204	

Note

NODF, BR, SR_Bin, robustness, and connectance are calculated on presence/absence networks. SR_Qua, WNODF, and modularity are calculated on abundance‐based networks. The results for the normalized degree are presented in the Appendix A.

Abbreviations: BR, discrepancy; NODF, nestedness index based on overlap and decreasing fill; SR_Bin, spectral radius calculated on binary (absence/presence) matrices; SR_Qua, spectral radius calculated on weighted (abundance) matrices; WNODF, NODF calculated on weighted matrices.

**Figure 2:**
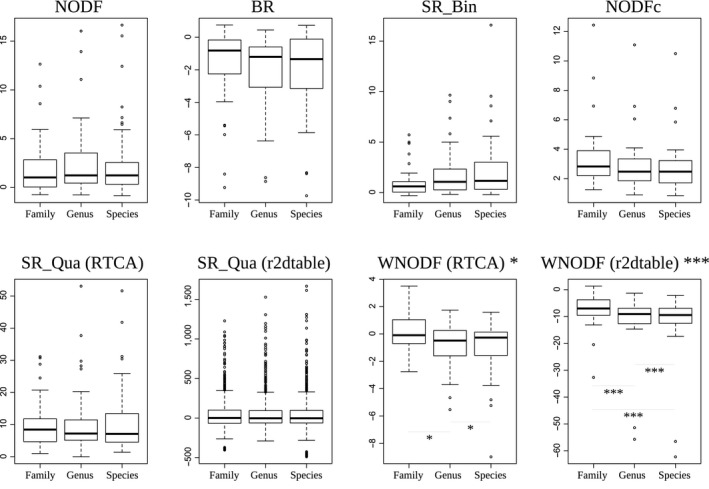
*Z*‐score distribution for nestedness indices for species‐, genus‐, and family‐determined matrices. BR, discrepancy; NODF, nestedness index based on overlap and decreasing fill; NODFc, NODF normalized according to Song et al.'s method (see Materials and Methods); SR_Bin, spectral radius calculated on binary (absence/presence) matrices; SR_Qua, spectral radius calculated on weighted (abundance) matrices; WNODF, NODF calculated on weighted matrices. 0.001 < *** < 0.01 < ** < 0.05; *Next to the index name reflects the result of the ANOVA test, on the graph itself reflects the results of the post hoc paired *t* test, with a Bonferroni correction. (RTCA) and (r2dtable) specify the results obtained through two different null models

We used the *Z*‐score (with two kinds of null models for the weighted indices) to take into account the difference in the matrix fills and sizes caused by the change in taxonomic resolutions, as well as another normalization by the maximal NODF (noted as NODF_c_). We compared nestedness *Z*‐scores and NODF_c_ values from one taxonomic level to another using a one‐way analysis of variance test. After this standardization, only the WNODF showed a significant effect of taxonomic level on its *Z*‐score value (Figure 2 and Table 3), with a higher level of significance using the r2dtable null model than the RTCA. NODF_c_ showed no influence of the taxonomic level on its values.

**Table 3:** Results of one‐way analysis of variance comparing *Z*‐score values (as well as NODF normalized according to Song et al. (2017)) at three different taxonomic resolutions

Indices	*df*	*F*	*p*‐Value	Post hoc test results
Nestedness				
NODF	2, 120	0.5698	.5672	
BR	2, 120	0.4745	.6233	
SR_Bin	2, 120	2.6813	.07258	
SR_Qua (RTCA)	2, 90	0.0729	.9297	
SR_Qua (r2dtable)	2, 90	0.3565	.7002	
WNODF (RTCA)	2, 90	3.0987	.04995	Genus and species do not differ significantly, while the two other comparisons are significantly different
WNODF (r2dtable)	2, 90	52.971	<2.2e−16	All levels differ significantly
NODF_c_	2, 104	0.9219	.4011	

Note

(RTCA) and (r2dtable) indicate which null model was used.

**Figure 3:**
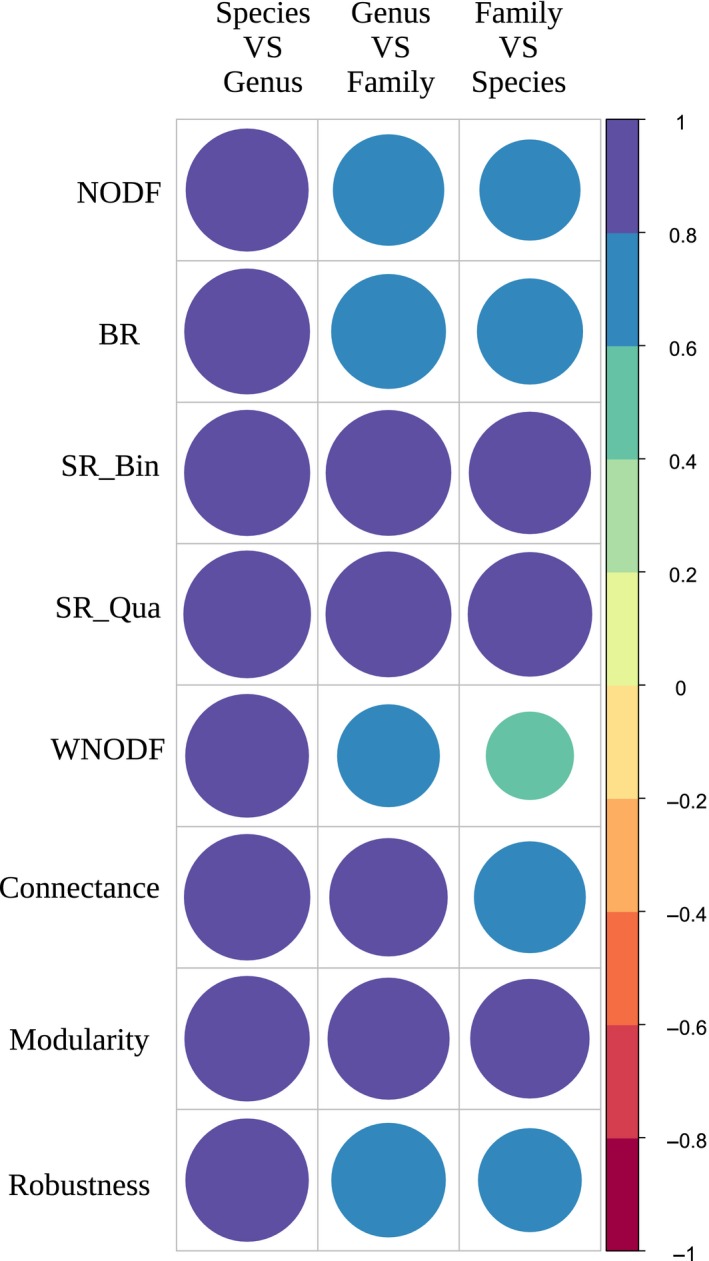
Correlation strength for all taxonomic levels and all indices. All correlations are significant. BR, discrepancy; NODF, nestedness index based on overlap and decreasing fill; SR_Bin, spectral radius calculated on binary (absence/presence) matrices; SR_Qua, spectral radius calculated on weighted (abundance) matrices; VS, versus; WNODF, NODF calculated on weighted matrices

While most untransformed indices for species‐, genus‐, and family‐determined matrices are significantly different, their values seem very strongly correlated among them. Using the Spearman's rho calculation, we observed that all tested index ranks showed a high positive correlation (Figure 3; for the results of the normalized degrees and motifs frequencies, see the Appendix A), indicating that the ranks of indices are well conserved at different taxonomic resolutions. All indices show a rank correlation superior to .8 between species and genus ranks, except for one motif frequency and certain quartile values of the normalized degree. This strong correlation becomes weaker for most indices as the taxonomic levels increase, particularly for comparisons with family versus species. Among the indices, the strongest options seem to be modularity and SR for nestedness, as they show a strong correlation (superior to .8) between ranks at the species, genus, and family levels for all possible comparisons. On the contrary, motif frequencies seem to be less reliable, as they show lower values for correlation between ranks than other indices, especially at the family versus species level, with most correlations being bellow .6 and some even below .35 (see the Appendix A for the full values).

## 4 DISCUSSION

Using plant–pollinator interaction networks from the literature, we showed that modifying the taxonomic resolution of these networks significantly changes the absolute values of the indices that describe their properties, except for two indices, namely the SR (both for binary and quantitative matrices) and robustness to species loss. If a standardization of the indices measuring nestedness is performed using the *Z*‐score, then three indices—NODF, BR, and SR for both binary and weighted matrices—are not significantly different at different taxonomic resolutions. Finally, the ranks of all indices are strongly conserved at different taxonomic resolutions, particularly between the species and genus levels.

For nestedness, we observed for both NODF and BR that the absolute values of these indices, but not the associated *Z*‐scores, are strongly modified by the change in taxonomic resolution. This result is in agreement with the work of Almeida‐Neto et al. (2008) who showed that NODF and BR are markedly affected by the matrix fill, a parameter that is modified when the taxonomic resolution is changed. However, this effect is no longer present after standardization using the *Z*‐score. Nevertheless, we observed that the absolute values of the SR for both binary and quantitative matrices are not modified by the change in taxonomic resolution, in accordance with the results of Strona and Fattorini (2014), which show an absence of relationship between the SR values and the filling of the matrices. We also showed that the normalization method proposed by Song et al. (2017) offers an NODF index robust to taxonomic resolution, which is in line with their own conclusions that NODF_c_ is independent from network number of rows, columns, and number of links, making it remarkably relevant to compare networks across studies or spatial gradients. Our results are also in agreement with an as yet unpublished study by Hemprich‐Bennett, Oliveira, Comber, Rossiter, and Clare, (2018) who also found that absolute measures of most of the metrics they tested (which includes NODF, robustness, connectance, but not SR or BR) vary according to the taxonomical level of the networks (both observed networks and networks deduced from metabarcoding data).

The main objective of our study was to determine whether it is possible to meaningfully estimate indices describing the characteristics of plant–pollinator interaction networks with a taxonomic resolution lower than the species. Our results suggest that it is indeed the case. To estimate nestedness, our results suggest that only the absolute values of SR indices are minimally impacted by changes in taxonomic resolution and should therefore probably be preferred when the objective is to compare nestedness levels for networks with a lower resolution than the species. Alternatively, it is possible to use NODF and BR after standardization using the *Z*‐score. For the other properties of the networks that we examined, namely connectance, modularity, normalized degree, and robustness to species loss, the absolute values of the indices cannot be directly compared at resolutions lower than the species level, but it is still possible to rank networks according to their values for these indices, because such ranks are well preserved when the level of taxonomic resolution changes. Motif frequencies do not present a unique pattern of sensitivity to taxonomic resolution. Indeed, some motif frequencies are significantly influenced by taxonomic resolution, while others are not. However, they all show a good preservation of the ranks between species and genus networks. We would advise not to use motif frequencies at a family level, though, as the correlation between ranks gets rather low (sometimes as low as 0.2).

Note, however, that whereas the taxonomic resolution lower than the species seems to allow us to characterize the properties of plant–pollinator interaction networks, it may make it more difficult to interpret these properties. One of the main objectives of the measurement of network properties is to make or test inferences about their underlying mechanisms. For example, Junker et al. (2013) showed that sets of plant traits such as phenology, floral reflectance, and morphology can predict plant–pollinator interactions and thus network structure. Similarly, Klumpers, Stang, and Klinkhamer (2019) showed that size matching between the pollinator proboscis length and the nectar tube depth is important in shaping plant–pollinator interactions. Such conclusions would be more difficult to reach when working above the species level. In the future, working on these levels would require careful consideration: Can functional traits be extended to the whole genus in that particular case? If this is not possible, then working on these levels could thus deprive us of a significantly explanatory variable. This means that while genus‐ and family‐level networks are usable and interpretable, they still entail a loss of information for future studies. For this reason, future studies need to consider the gain in network explicitness versus the loss of information before choosing to work at the genus or family level.

Our results support the relevance of citizen science for ecological research. The major strengths of citizen science programs lie in their ability to conduct studies at large geographic scales and on private properties, which are usually impossible to perform with traditional field research (Dickinson et al., 2010), although these are often at the price of a lower taxonomical precision. Here, we showed that datasets with a taxonomic resolution lower than the species level can be used to estimate the properties of networks assembled at the same resolution, even if it is lower than the species. However, plant–pollinator interaction data produced by citizen science are probably characterized by relatively low sampling completeness, because detecting all the species interactions is extremely labor‐intensive (Chacoff et al., 2012), which can have an effect on the estimated properties of the networks. For the indices that we studied, Rivera‐Hutinel, Bustamante, Marín, and Medel (2012) showed that nestedness, modularity, and robustness to species loss are little affected by sampling completeness, whereas connectance is very sensitive to low sampling. In conclusion, sets of plant–pollinator networks produced by citizen science, frequently characterized by low taxonomic resolution and low sampling efforts, are probably best analyzed by calculating their nestedness with SR (or NODF and BR after standardization using the *Z*‐score) and their robustness with species loss, and then ranking them according to their modularity.

Our work confirms that we can use protocols with only genus‐ or family‐level data and still use network‐level analyses of plant–pollinator interactions. An interesting complement would be to study the same question for other kinds of mutualistic networks such as ant–plant networks or even for other kinds of interaction networks such as food webs.

## AUTHORS’ CONTRIBUTIONS

CBG and EB conceived the ideas and designed methodology. ER collected the data. ER, CBG, and EB analyzed the data. ER led the writing of the manuscript. All authors contributed critically to the drafts and gave final approval for publication.

## Data Availability

The networks used in this analysis are available at the Interaction Web Database (https://www.nceas.ucsb.edu/interactionweb/resources.html#plant_pollinator), which collects datasets from authors publishing their work. Some of them, however, were not used due to the difficulty of treatment. The list of articles used in our study is available in Supplementary Materials. The complete script used to obtain the result and diverse networks is accessible here: https://gitlab.com/EstelleRenaud/taxonomic_influence_network_properties

## Appendix A

**Table A1:** Results of one‐way analysis of variance comparing normalized degree (ND) values and motif frequencies at three different taxonomic resolutions

Indices	*df*	*F*	*p*‐Value	Post hoc test result
Normalized degrees for pollinators—lower limit of 1st quartile	2, 120	5.7164	.004252	All levels differ significantly
ND_high_2	2, 120	10.92	4.396e−05	All levels differ significantly
ND_high_3	2, 120	13.129	6.977e−06	All levels differ significantly
ND_high_4	2, 120	18.155	1.293e−07	All levels differ significantly
ND_high_5	2, 120	9.2779	.0001792	All levels differ significantly
ND_low_1	2, 120	20.594	2.046e−08	All levels differ significantly
ND_low_2	2, 120	15.191	1.316e−06	All levels differ significantly
ND_low_3	2, 120	11.922	1.893e−05	All levels differ significantly
ND_low_4	2, 120	18.947	7.06e−08	All levels differ significantly
ND_low_5	2, 120	13.857	3.85e−06	All levels differ significantly
Motif 1	2, 120	1	.3709	
Motif 2	2, 120	1.1355	.3247	
Motif 3	2, 120	1.1355	.3247	
Motif 4	2, 120	0.112	.8942	
Motif 5	2, 120	2.1753	.118	
Motif 6	2, 120	9.3845	.0001634	All levels differ significantly
Motif 7	2, 120	2.5995	.07849	
Motif 8	2, 120	0.1919	.8256	
Motif 9	2, 120	0.2043	.8155	
Motif 10	2, 120	1.7185	.1837	
Motif 11	2, 120	9.5841	.0001376	All levels differ significantly
Motif 12	2, 120	5.4115	.005621	All levels differ significantly
Motif 13	2, 120	0.0374	.9633	
Motif 14	2, 120	0.9314	.3968	
Motif 15	2, 120	3.4154	.03609	Species and genus do not differ significantly, while the two other comparisons are significantly different
Motif 16	2, 120	4.8719	.00924	Species and genus do not differ significantly, while the two other comparisons are significantly different
Motif 17	2, 120	3.4329	.0355	All levels differ significantly

Note

Results are compiled for pollinator (“high”) and plant (“low”) taxa, for each bound of the quartile values (1–5).

**Table A2:** Results of Spearman's rho correlation tests comparing the relative values of all networks between two taxonomic levels, for each index

Index	Species vs Genus		Genus vs Family		Species vs Family	
	Rho estimate	*p*‐Value	Rho estimate	*p*‐Value	Rho estimate	*p*‐Value
NODF	.914	<2.2e−16	.751	1.611e−07	.617	2.62e−05
WNODF	.926	1.541e−08	.6375	.0001612	.467	.008709
BR	.959	<2.2e−16	.797	4.478e−10	.678	1.093e−06
SR Binary	.962	<2.2e−16	.956	<2.2e−16	.903	<2.2e−16
SR Weighted	.988	<2.2e−16	.960	<2.2e−16	.939	<2.2e−16
Connectance	.968	<2.2e−16	.948	2.705e−12	.754	1.224e−08
Modularity	.952	<2.2e−16	.900	1.343e−07	.863	3.964e−07
Robustness	.921	<2.2e−16	.793	6.482e−10	.650	4.219e−06
ND_high_1	.994	<2.2e−16	.828	2.357e−11	.823	3.734e−11
ND_high_2	.904	5.834e−16	.819	6.084e−11	.681	9.452e−07
ND_high_3	.933	<2.2e−16	.776	2.533e−09	.712	1.786e−07
ND_high_4	.914	<2.2e−16	.824	3.773e−11	.724	8.565e−08
ND_high_5	.886	1.436e−14	.693	5.144e−07	.634	8.632e−06
ND_low_1	.923	<2.2e−06	.741	2.984e−08	.641	6.3e−06
ND_low_2	.890	6.742e−15	.762	7.313e−09	.726	7.695e−08
ND_low_3	.940	<2.2e−16	.779	1.947e−09	.666	2.013e−06
ND_low_4	.936	<2.2e−16	.853	1.453e−12	.721	1.039e−07
ND_low_5	.910	<2.2e−16	.734	4.871e−08	.605	2.791e−05
Motif 1	NA	NA	NA	NA	NA	NA
Motif 2	.935	<2.2e−16	.641	6.319e−06	.575	8.341e−05
Motif 3	.935	<2.2e−16	.641	6.319e−06	.575	8.341e−05
Motif 4	.935	<2.2e−16	.756	1.356e−07	.685	1.742e−06
Motif 5	.859	<2.2e−16	.661	4.746e−06	.633	1.404e−05
Motif 6	.906	<2.2e−16	.702	3.201e−07	.558	.0001514
Motif 7	.930	<2.2e−16	.614	1.989e−05	.578	7.461e−05
Motif 8	.927	<2.2e−16	.793	6.482e−10	.735	4.315e−08
Motif 9	.937	<2.2e−16	.703	8.524e−07	.631	1.542e−05
Motif 10	.921	<2.2e−16	.402	.009617	.399	0.01027
Motif 11	.913	<2.2e−16	.418	.006511	.285	0.07137
Motif 12	.953	<2.2e−16	.708	2.208e−07	.608	2.464e−05
Motif 13	.900	<2.2e−16	.585	8.009e−05	.435	.004839
Motif 14	.788	3.833e−08	.617	1.742e−05	.519	.0005086
Motif 15	.807	6.773e−09	.742	2.875e−08	.508	.0006918
Motif 16	.815	8.911e−11	.830	2.023e−11	.644	5.625e−06
Motif 17	.933	<2.2e−16	.606	2.661e−05	.560	.0001424

Abbreviation: BR, discrepancy; ND, normalized degree compiled for pollinator (“high”) and plant (“low”) taxa, for each bound of the quartile values (1–5); NODF, nestedness index based on overlap and decreasing fill; SR_Bin, spectral radius calculated on binary (absence/presence) matrices; SR_Qua, spectral radius calculated on weighted (abundance) matrices; WNODF, NODF calculated on weighted matrices.
