# Supplementary material for: Influence of taxonomic resolution on mutualistic network properties
Source: Ecol Evol. 2020 Mar 6;10(7):3248–59. doi: 10.1002/ece3.6060 (PMC7140996; doi:10.1002/ece3.6060)
Supplement: Supplementary file 1 [file ECE3-10-3248-s001.docx]

| **Dataset** | **Habitat type** | **Location** | **Data type** | **No. plants** | **No. pollinators** | **DOI** |
| --- | --- | --- | --- | --- | --- | --- |
| Arroyo et al. (1985) (three networks) | Andean scrub | Chile | Binary | 87 | 98 | 10.1007/ BF00983305 |
|  | Andean scrub | Chile | Binary | 43 | 62 |  |
|  | Andean scrub | Chile | Binary | 41 | 28 |  |
| Barrett & Helenurm (1987) | Boreal forest | Canada | Individuals caught | 12 | 102 | 10.1139/ b87-278 |
| Bezerra et al. (2009) | Caatinga (semi-arid vegetation) | Pernambuco State, Brazil | No. of visits | 13 | 13 | 10.1111/ j.1365-2656.2009.01567.x |
| Clements & Long (1923) | Montane forest and grassland | USA | Binary | 96 | 276 |  |
| Dupont et al. (2003) | High-altitude desert | Tenerife, Canary Islands | Binary | 11 | 38 | 10.1034/ j.1600-0587.2003.03443.x |
| Elberling & Olesen (1999) | Alpine subarctic community | Sweden | No. of visits | 23 | 118 | 10.1111/ j.1600-0587.1999.tb00507.x |
| Hocking (1968) | Arctic community | Canada | Binary | 29 | 86 | 10.2307/ 3565022 |
| Kaiser-Bunbury et al. (2009) (two webs) | Heathland with introduced plants | Mauritius | Rates | 135 | 74 | 10.1016/ j.ppees.2009.04.001 |
|  | Heathland without introduced plants | Mauritius | Rates | 100 | 64 |  |
| Kaiser-Bunbury et al. (2017) (eight webs) | Seychelles restoration networks | Seychelles | No. of visits and visitation frequency | 62 | 144 | 10.1038/ nature21071 |
|  |  |  |  | 56 | 144 |  |
|  |  |  |  | 75 | 144 |  |
|  |  |  |  | 58 | 144 |  |
|  |  |  |  | 53 | 144 |  |
|  |  |  |  | 55 | 144 |  |
|  |  |  |  | 68 | 144 |  |
|  |  |  |  | 67 | 144 |  |
| Kevan (1972) | High arctic | Canada | No. of visits | 32 | 115 | 10.2307/ 2258569 |
| Inouye & Pyke (1988) | Montane forest | Australia | Individuals caught | 42 | 91 | 10.1111/ j.1442-9993.1988.tb00968.x |
| McMullen (1993) | Multiple communities | Galapagos Islands | Binary | 106 | 54 |  |
| Medan et al. (2002) (two webs) | Xeric scrub | Argentina | Binary | 21 | 45 | 10.1080/ 15230430.2002. 12003490 |
|  | Woody riverine vegetation and xeric scrub | Argentina | Binary | 23 | 72 |  |
| Memmott (1999) | Meadow | UK | Frequency of visits | 25 | 79 | 10.1046/ j.1461-0248.1999.00087.x |
| Mosquin (1967) | Arctic community | Canada | Individuals caught | 11 | 18 |  |
| Motten (1986) | Deciduous forest | USA | No. of visits | 10 | 12 | 10.2307/ 2937269 |
| Olesen et al. (2002) (two webs) | Coastal forest | Mauritius Island | No. of visits | 14 | 13 | 10.1046/ j.1472-4642.2002.00148.x |
|  | Rocky cliff and open herb community | Azores Islands | No. of visits | 10 | 12 |  |
| Ramirez & Brito (1992) | Palm swamp community | Venezuela | Binary | 33 | 53 | 10.1111/ j.1095-8339.1992.tb00294.x |
| Santos et al. (2010) | Caatinga (semi-arid vegetation) | Brazil | Binary | 51 | 25 | 10.1051/apido/ 2009081 |
| Schemske et al. (1978) | Maple-oak woodland | USA | No. of visits | 7 | 32 | 10.2307/ 1936379 |
| Small (1976) | Peat bog | Canada | Individuals caught | 13 | 34 |  |
| Vázquez & Simberloff (2002) (eight webs) | Evergreen montane forest | Argentina | No. of visits | 10 | 29 | 10.1086/ 339991 |
|  | Evergreen montane forest | Argentina | No. of visits | 9 | 33 |  |
|  | Evergreen montane forest | Argentina | No. of visits | 9 | 27 |  |
|  | Evergreen montane forest | Argentina | No. of visits | 10 | 29 |  |
|  | Evergreen montane forest | Argentina | No. of visits | 8 | 35 |  |
|  | Evergreen montane forest | Argentina | No. of visits | 8 | 26 |  |
|  | Evergreen montane forest | Argentina | No. of visits | 7 | 24 |  |
|  | Evergreen montane forest | Argentina | No. of visits | 8 | 27 |  |
